# Temporal assessment of the medicinal plants trade in public markets of the state of Paraíba, northeastern Brazil

**DOI:** 10.1186/s13002-021-00496-3

**Published:** 2021-12-19

**Authors:** Ezequiel da Costa Ferreira, Reinado Farias Paiva de Lucena, Rainer W. Bussmann, Narel Y. Paniagua-Zambrana, Denise Dias da Cruz

**Affiliations:** 1 Laboratório de Ecologia Terrestre, Dept. de Sistemática E Ecologia, Centro de Ciências, Exatas e da Natureza, Universidade Federal da Paraíba, João Pessoa, PB 58051-900 Brazil; 2 Programa de Pós-Graduação Em Desenvolvimento E Meio Ambiente, PRODEMA, Universidade Federal da Paraíba, João Pessoa, PB 58051-900 Brazil; 3 Instituto de Biociências, Universidade Federal Do Mato Grosso Do Sul, Campo Grande, 79070-900 Brazil; 4 Department of Ethnobotany, Institute of Botany and Bakuriani Alpine Botanical Garden, Ilia State University, 1 Botanical Str., 0105 Tbilisi, Georgia

**Keywords:** Urban ethnobotany, Caatinga, Atlantic Forest, Similarity analysis, Relative importance

## Abstract

**Background:**

Open and public markets are the main providers of medicinal plants in urban environments. The present study evaluated the medicinal plants sold in public markets in different municipalities in the mesoregions of the state of Paraíba, northeast of Brazil, and the possible variations in the supply of these plants in the markets over the course of a year.

**Methods:**

Interviews with medicinal plant traders were conducted in four mesoregions of different climatic and phytophysiognomic characteristics (ranging from Caatinga to Atlantic Forest). The versatility of the species sold was elucidated using the relative importance (RI) index, and the set of species sold by each informant in each mesoregion was compared with each other by one-way Anosim  and by the analysis of main coordinates.

**Results:**

Thirty-five plant traders identified 163 medicinal plant species (151 genders and 76 families) and more 17 non identified species. The most frequent families were Fabaceae (19 species), Asteraceae (12), Lamiaceae (11), and Myrtaceae (6). *Punica granatum*, *Zingiber officinale*, and *Myracrodruon urundeuva* were the species with the highest RI. The analysis of similarity showed distinct differences between the Sertão and all other mesoregions. The Agreste, an ecotone area, was also the area where more species of other regions was found. The absence of 88 species in at least one of the trading locations at some stage of the fieldwork was recorded.

**Conclusions:**

The presence and absence of the commercialized species do not seem to be related to the period of the year or the mesoregion. There were differences in the inventory of plants commercialized in markets in recent years. We identified an intermediate zone of knowledge and use of species commercialized between the studied localities.

## Background

The knowledge about and use of medicinal plants are themes that remain one of the main study topics in ethnobotany. Many recent studies in Brazil and around the globe have recorded the knowledge and use of medicinal plants, in both rural [e.g., 1–8] and urban areas [e.g., 9–15].

In urban areas, open air and public markets are some of the main sources of medicinal plants. There it is possible to find these products traded and to observe variations with regard to both the plant parts sold and the inventory of available species over time. Several studies observed a predominance of the medicinal use of non-permanent plant structures, such as leaves [8, 16, 17]. Some studies in Brazil showed that overall, changes in the list of traded species occurred as a function of the temporal availability or the demand for certain species in the market [10, 18–20]. In other cases, it was possible to observe a relatively constant inventory of medicinal plants, with the inclusion of some new species over time, e.g., in La Paz, Bolivia [21], in both the short and medium term [21]. When observing broader temporal contexts, the changes in the inventory of medicinal plants can become more evident, as observed in Peru, where the local pharmacopeia has been changing since the colonial period [22].

The climate and the predominant phytophysiognomy (the main plant physiognomy or vegetation cover in a region) in a given region can also influence the set of traded species or the plant parts used. From this perspective, the more ample use of permanent plant structures such as bark and roots has been often documented from drier areas, such as the Caatinga [23–25] (semiarid region of Brazil), as well as in Savannah and Desert areas in Africa [26–28]. In humid areas, it has been more common to observe a greater use of leaves, such as in the Atlantic Forest [29] and the Amazon [30], as well as in rainforest areas in Asia [17, 31, 32].

In addition to the availability of the specific plant parts used, several other factors can influence the availability of traded products, such as seasonality [18, 20], demand [18], and local environmental factors [33]. Repeated sampling can be useful to identify and understand these variations in the medicinal plant products available for trade [10, 33].

Variations in the availability of traded plants have also been observed when comparing different public markets, even in nearby areas [21] or in long-term comparisons with previous studies [19, 21]. Another issue that can influence the trade and use of medicinal plants is globalization, especially due to the increase and use of social and digital media to advertise and trade these products [34], and the medicinal plant trade in the international context [13].

The present study aimed to assess the medicinal plants traded in the public markets of municipalities in four mesoregions of the state of Paraíba, northeastern Brazil, each with its independent climatic and phytophysiognomy characteristics, but with two well-defined biomes: the Caatinga semiarid region, and the Atlantic Forest humid region. The assessment was performed by documenting the species used and the possible variations in their availability in the markets throughout the year. We hypothesized that there would be a greater influence of seasonality on the availability of medicinal plants for trade in the interior of the state, a region with a semiarid climate, than in the coast, which is subject to higher humidity over the year, especially with respect to native species. The hypothesis assumes that in areas where rainfall is more uniformly distributed throughout the year, such as the coast, vegetative structures of the native flora also maintain certain uniformity throughout the year, such as perennial species that keep the leaves. So, the inventory of the plants is often higher and more parts of the plants can be found in the markets. On the other hand, in the interior of the state, characterized as a semiarid region, rainfall is seasonal and generally concentrated in specific periods of the year. In this region, deciduous plants are more common and structures such as the leaves can be shed during the dry season, and thus, other vegetative structures are used by the community. Thus, the interior of the state of Paraíba, with a semiarid climate, would have a more marked influence of seasonality on the use of plants.

## Materials and methods

### Study area

**Fig. 1:**
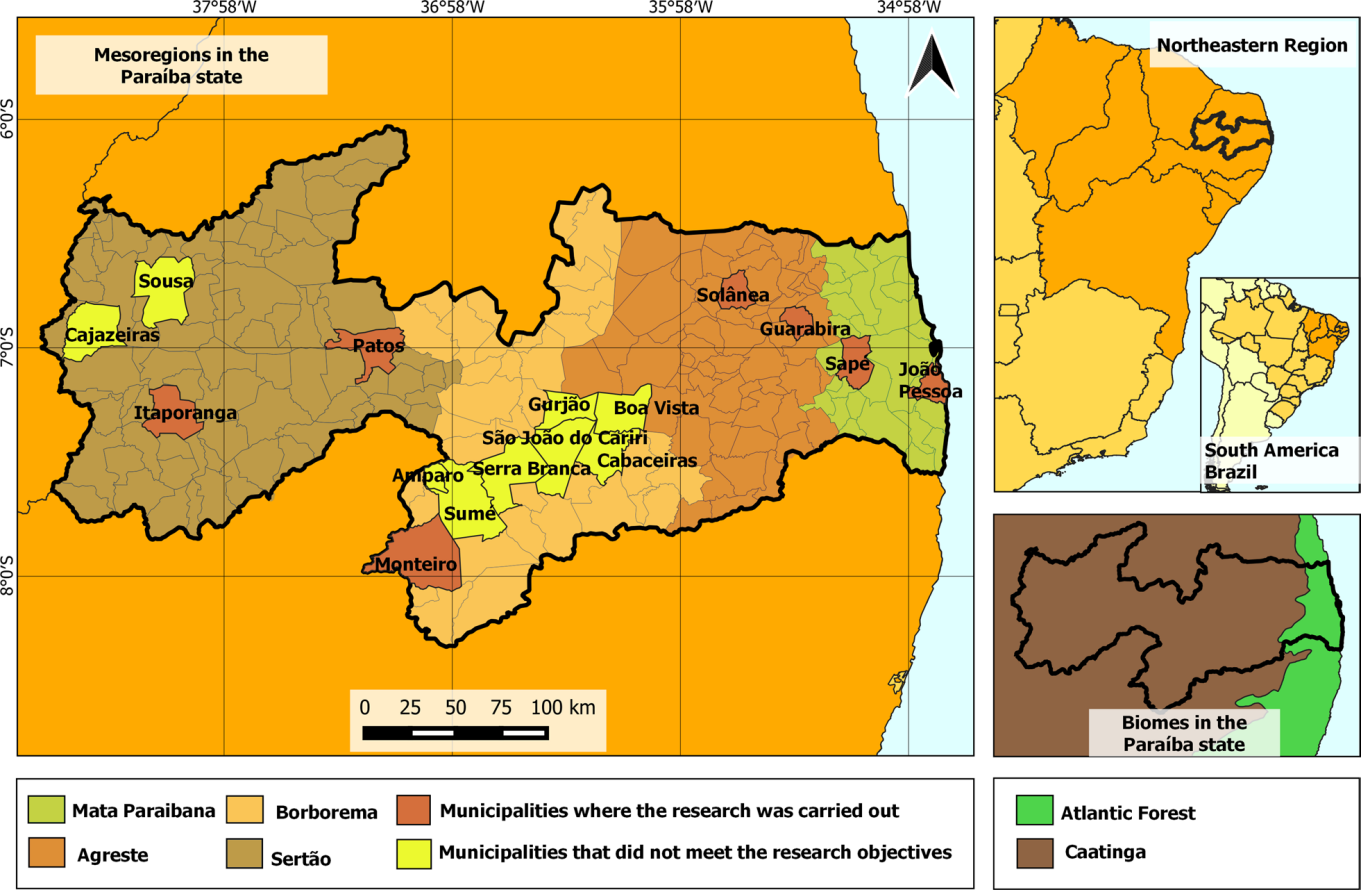
Map of the state of Paraíba, Brazil, highlighting the studied municipalities, the municipalities where it was impossible to conduct the research, the four mesoregions of the state, and the predominance areas of the biomes

The study was conducted in public markets of seven municipalities of Paraíba, distributed in the four mesoregions of the state: João Pessoa and Sapé (Mata mesoregion), Guarabira and Solânea (Agreste mesoregion), Monteiro (Borborema mesoregion), and Patos and Itaporanga (Sertão mesoregion). The predominant phytophysiognomy in the municipalities of João Pessoa and Sapé is Atlantic Forest, while Caatinga vegetation predominates in the remaining municipalities (Fig. 1). These mesoregions are defined by socioeconomic and environmental characteristics, showing marked climatic variations. In 2017, when our fieldwork was already in progress, the IBGE (Brazilian Institute of Geography and Statistics) changed the division of the geographic regions of Brazil, modifying the configuration and the classification as mesoregions and microregions into intermediate regions and immediate regions, respectively [35]. For this study, we adopted the mesoregion and microregion classification, because this classification allows better visualization of the different phytophysiognomies of the state, considering that the new Regional Division of Brazil is more focused on socioeconomic aspects [35], not highlighting the environmental differences.

The Mata Paraibana mesoregion is characterized by a hot and humid climate [36] with three climate types according to the classification by Köppen: Aw (tropical, with a dry season in winter), Am (high rainfall), and As (hot and humid tropical, with dry winter) [37]. The Mata Paraibana shelters what little is left of the Atlantic Forest in Paraíba, most which has been destroyed by anthropogenic impact, especially the expansion of sugarcane production. This zone also incorporates beaches, plateaus, floodplains, and estuaries [36].

The Agreste comprises a transition area between the humid and the semiarid climate [36], belonging to the Köppen climate types As and Bsh (hot semiarid) [37]. Its vegetation represents also a transition area between the Atlantic Forest and the Caatinga [36].

The Borborema comprises the central area of the state of Paraíba, located in the geomorphological unit of the Borborema Plateau [36]. It shows the Köppen climate types As and Bsh [37], with the lowest rainfall levels in the state, and salty, thin, and rocky soils.

The Sertão comprises several depressions, with a semiarid climate, and a vegetation characteristic of the Caatinga [36]. This area is divided between the Köppen climate types Bsh (hot semiarid) and As (hot and humid tropical, with dry winter) [37].

Before beginning the interviews, previous visits were made to identify locations that met the objectives of the study. It was impossible to conduct the research in some of the visited municipalities (Fig. 1), either because they did not have a public market or because there were no medicinal plants traded in the market.

### Data collection

**Fig. 2:**
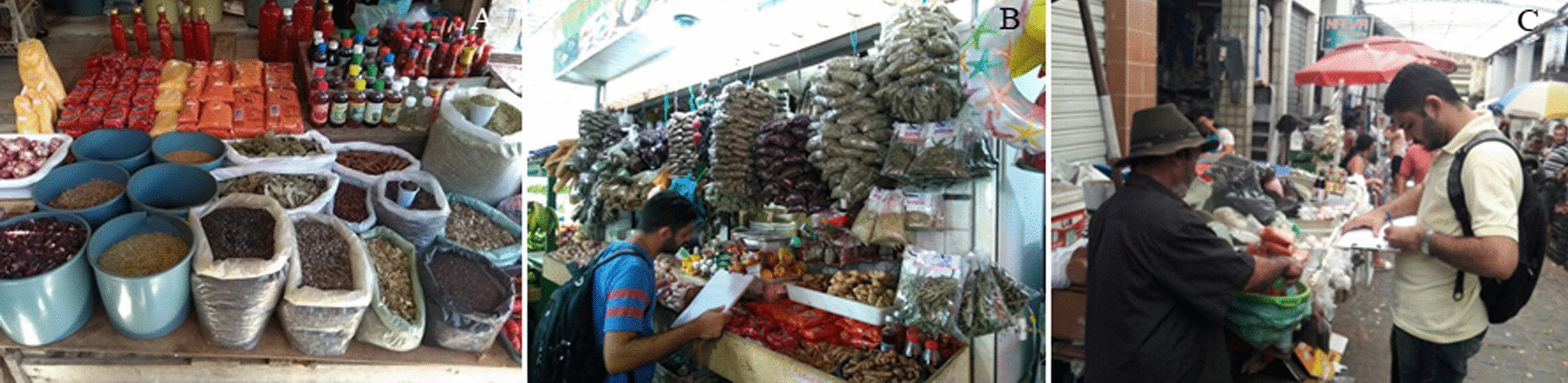
Posts of medicinal plants in public markets of Paraíba state, northeast of Brazil. **A** Central market, João Pessoa; **B** Mangabeira market, João Pessoa; **C** public market, Guarabira

Visits were made to the trading locations of medicinal plants in the public markets of the municipalities (Fig. 2). After initial contact to properly present and explain the purposes of the study, the medicinal plant traders in the markets were invited to sign the Free Consent Form (TCLE) required by the National Health Council through the Research Ethics Committee (Resolution 466/12) (Protocol: 82943618.0.0000.5188) and to participate in the research. In the countryside of the state, once a week the movement of commerce is intense and popularly known as “fair day.” The interviews were conducted, preferably, on days with less movement in the markets, avoiding the “fair day,” when traders have to pay more attention to their customers.

The free list technique was used based on the following question: “Which medicinal plants do you sell?” Subsequently, for each plant, details on their origin (was a plant from the local vegetation? did it come from other regions? was it imported?), considering both native and exotic species, and registering their applications, properties, preparation, parts used, and contraindications. In the context of this study, the stem was considered as both the shoot and its subterraneous structures (rhizome and bulb), when present. Repeat interviews were performed each trimester during one year (from August 2017 to July 2018), in order to evaluate if a species was either absent or added in relation to the previous periods. During each interview, a list of all plants available in a specific trade location was compiled, and further details were obtained on the use of each species.

The identification of the traded species was made by acquiring fertile specimens in the markets and collecting the cited species in the field when possible, and then comparing the material with the corresponding literature. At the end of each interview, samples of material were purchased from each trader, as a way of rewarding him/ her and strengthening the ties between researcher and interviewee. The names and families of the species were confirmed using REFLORA (Flora do Brasil 2020) [38] and the Missouri Botanical Garden database (Tropicos) [39]. The herborized plants were sent to the Herbarium Jaime Coelho de Moraes (EAN) of the Federal University of Paraíba (UFPB), Center of Agricultural Sciences (CCA), for confirmation of the identification and incorporation into the plant collection.

### Data analysis

The therapeutic indications mentioned by the informants were classified according to body systems (categories defined by the WHO for each property) [40]. The BS (number of body systems) and PH (number of pharmacological properties) for each species were calculated according to Bennett and Prance [41], with the following equations:

$${\text{BS}} = {\text{BSS}}/{\text{BSVS}}$$

where BS refers to the number of body systems, resulting from the division of the number of body systems treated by a given species (BSS) by the total number of body systems treated by the most versatile species (BSVS), considering as the most versatile the species that obtained the greatest diversity of body systems.

The following equation was used for the PH:

$${\text{PH}} = {\text{PHS}}/{\text{PHVS}}$$

where NP is the number of properties, resulting from the division of the number of properties attributed to a given species (PHS) by the number of properties attributed to the most versatile species (PHVS), considering as the most versatile the species that obtained the highest number of properties.

Subsequently, also based on Bennett and Prance [41], the relative importance (RI) of each species was calculated by the following equation:

$${\text{RI}} = {\text{BS}} + {\text{PH}}.$$

This method highlights the most versatile species or those with the greatest diversity of uses. It consists of a quantitative method that is not directly influenced by the number of citations for a given species but rather by the diversity of applications inferred to a species. The maximum value obtained is 2; the closer to this value is the RI of the plant, the greater its versatility, also considering that the RI of the species is high when ≥ 1.

The one-way ANOSIM permutation test was used to assess the degree of similarity of the species used between mesoregions (Bray–Curtis distance and 9999 permutations). This test produces an R result that ranges from − 1 to + 1, which may indicate no significant difference between groups (R < 0.25), while values between 0.25 < R < 0.5 indicate some data similarity and values of R > 0.75 indicate different results, with total difference when R = 1. Principal coordinates analysis (PCoA) using the Bray–Curtis distance was employed to generate a graph representing these differences between the cited species. The software Past 3.22 was used in the analyses. Data tabulation in the software was made based on the presence and absence of matrix in binary code, in which 1 represents the presence of the species considered in the trade location and 0 represents the absence.

## Results

All medicinal plant traders found in the markets that agreed to participate in the survey were interviewed. Only two traders refused to participate (1 in Zona da Mata and 1 in Sertão). Traders who were not found at the tends throughout all the research period (one year and quarterly monitoring) were excluded from the sample: Two traders in the Agreste and one in the Sertão were excluded. A total of 35 traders were interviewed (13 in the Mata Paraibana; 10 in the Agreste; 4 in the Borborema; 8 in the Sertão). The age of the traders ranged between 23 and 81 years, and 54.3% were men and 45.7% were women.

### Commercialized species, used category, and plant parts traded

A total of 163 species were identified at least to genus level, belonging to 151 genera and 76 families. Seventeen species remained unidentified. The most common families were Fabaceae (19 species), Asteraceae (12), Lamiaceae (11), and Myrtaceae (6) (Table 1).

A high relative importance value was recorded (RI ≥ 1) for 32 species, among which 11 were native to Brazil and 21 were exotic (Table 2). The species most often found in the markets were *Foeniculum vulgare* Mill. (*erva doce;* found in 35 posts, IR = 1.59), *Pimpinella anisum* L. (*erva doce;* 34, 1.59), *Peumus boldus* Molina *(boldo do Chile;* 33, 1.13), *Matricaria chamomilla* L. (*camomila;* 31, 1.17), all exotic species. The species that obtained the highest RI values were *Punica granatum* L. (*romã*; RI = 2), *Zingiber officinale* Roscoe (*gengibre*; 1.78), and *Myracrodruon urundeuva* Allemão (*aroeira*; 1.69) (Tables 1 and 2). Despite the high RI value observed, these species were registered in less than a half of the trade points, except *Z. officinale* which occurred in 23 points.

**Fig. 3:**
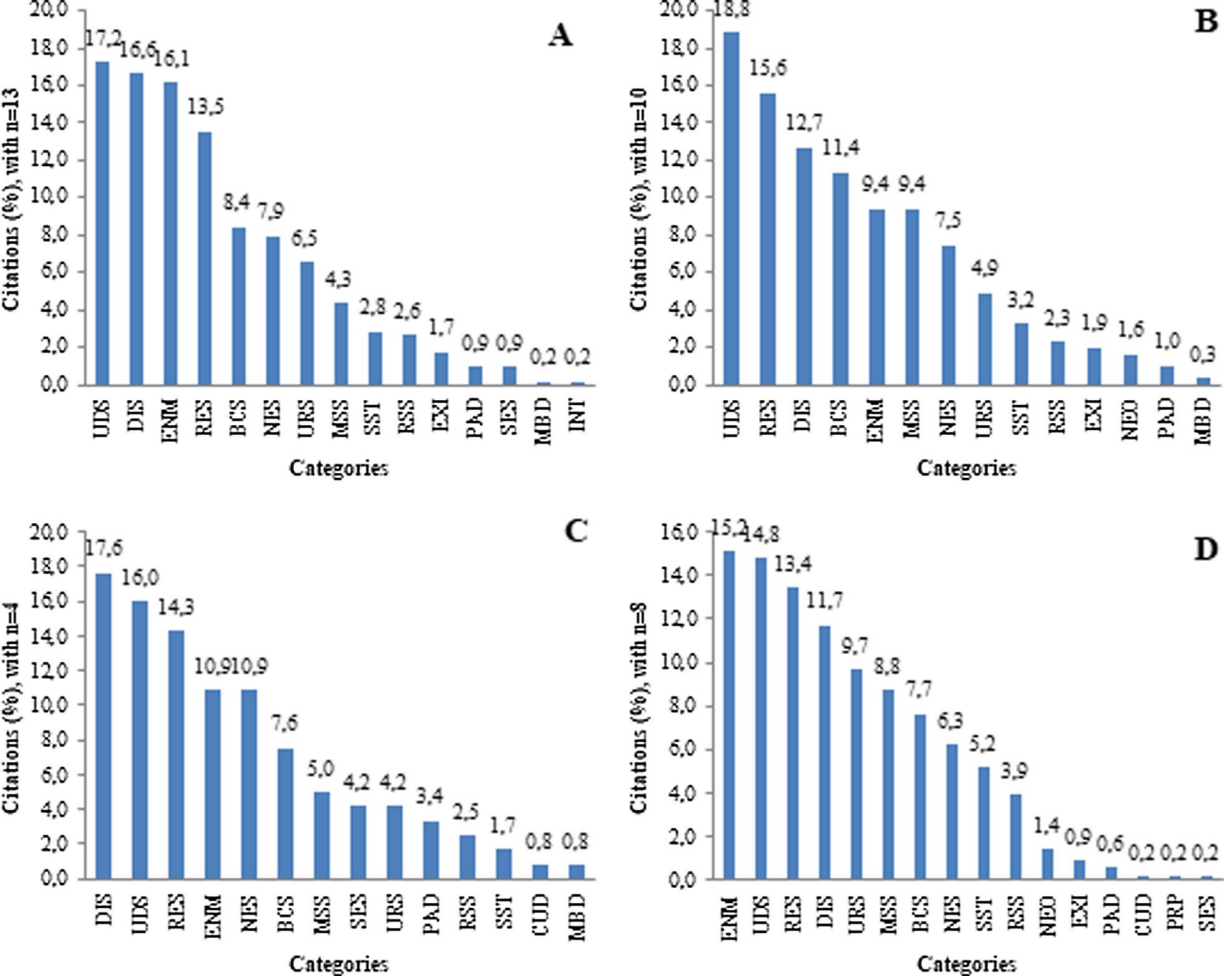
Percentage of citations for each category of medicinal use in public markets of Paraíba, northeastern Brazil. n = number of informants in the mesoregion. BCS = blood and cardiovascular system; CUD = cultural diseases; DIS = digestive system; ENM = endocrine system, nutrition, and metabolism; EXI = external injuries; INT = intoxication; MBD = mental and behavioral diseases; MSS = musculoskeletal system; NEO = neoplasms; NES = nervous system; PAD = parasitic diseases; PRP = pregnancy and parturition; RES = respiratory system; RSS = reproductive system and sexual health; SES = sensory system; SST = skin and subcutaneous tissue; UDS = unspecified diseases and symptoms; URS = urinary system. **A** Mata, **B** Agreste, **C** Borborema, **D** Sertão

The categories with the highest number of citations for each mesoregion were: unspecified diseases and symptoms; digestive system; endocrine system, nutrition, and metabolism; and respiratory system, with varying prevalence according to each mesoregion (Fig. 3). On the other hand, there was a variation in the categories with low number of citation among the mesoregions. Sensory system was more cited in the Borborema mesoregion than in others mesoregions. (In Agreste, this category was not cited by the interviewers.) Skin and subcutaneous tissue were less observed in the Borborema and, however, were frequent in the others mesoregions (Fig. 3).

**Fig. 4:**
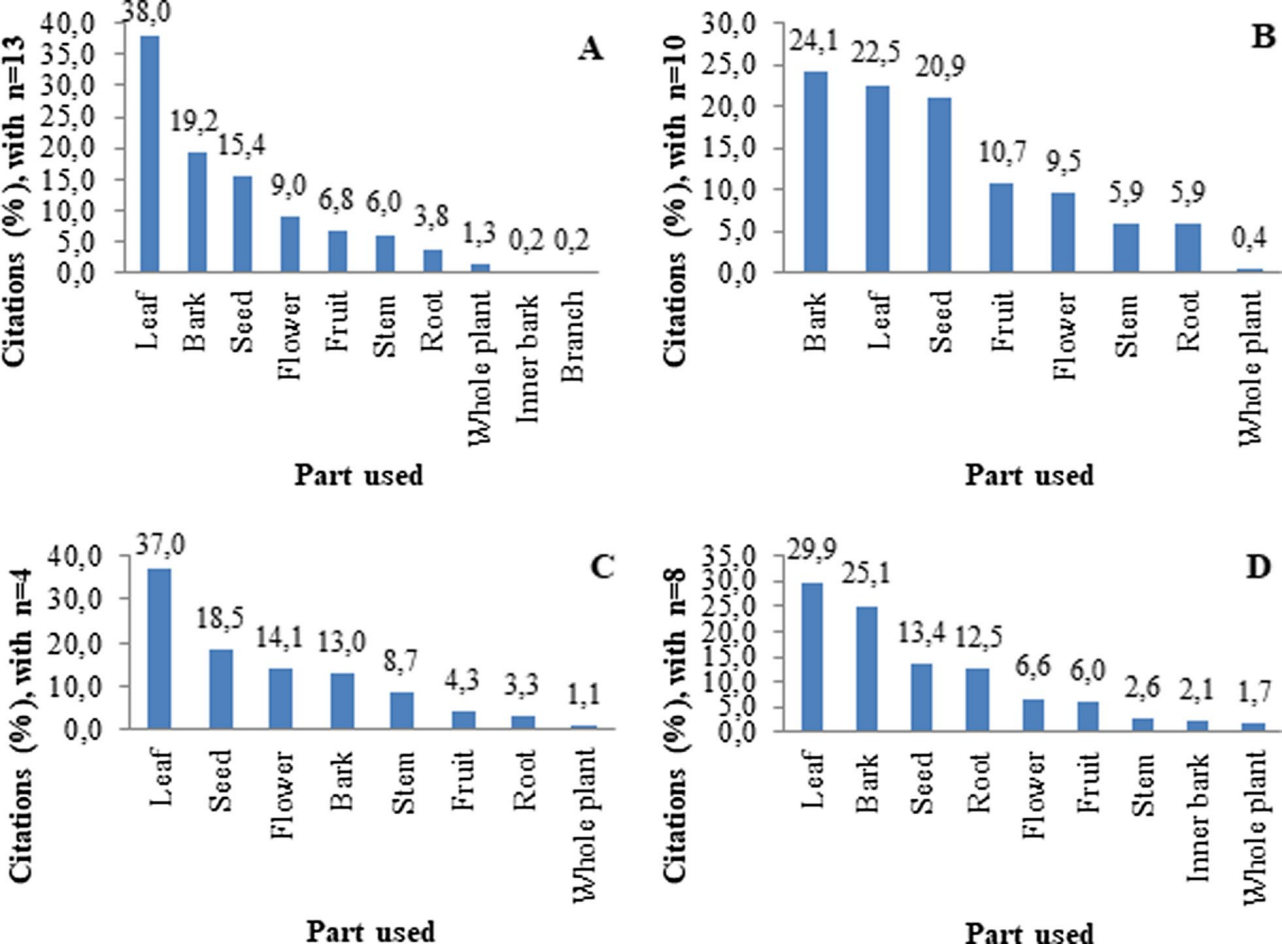
Percentage of citations for the plant parts traded for medicinal use in public markets of Paraíba, northeastern Brazil. n = number of informants in the mesoregion. **A:** Mata, **B:** Agreste, **C:** Borborema, **D:** Sertão

The plant parts traded most commonly for medicinal use were leaves, bark, and seeds, varying only with regard to their prevalence in each mesoregion (Fig. 4). The use of flowers stood out in the Borborema region (Fig. 4). The use of the bark predominates in the Agreste mesoregion, while leaves were the most traded plant structure in the remaining mesoregions. The bark commercialization also varied among the mesoregions; it was more common in the Zona da Mata and Borborema than in the Agreste and Sertão.

### Similarity between the mesoregions

**Fig. 5:**
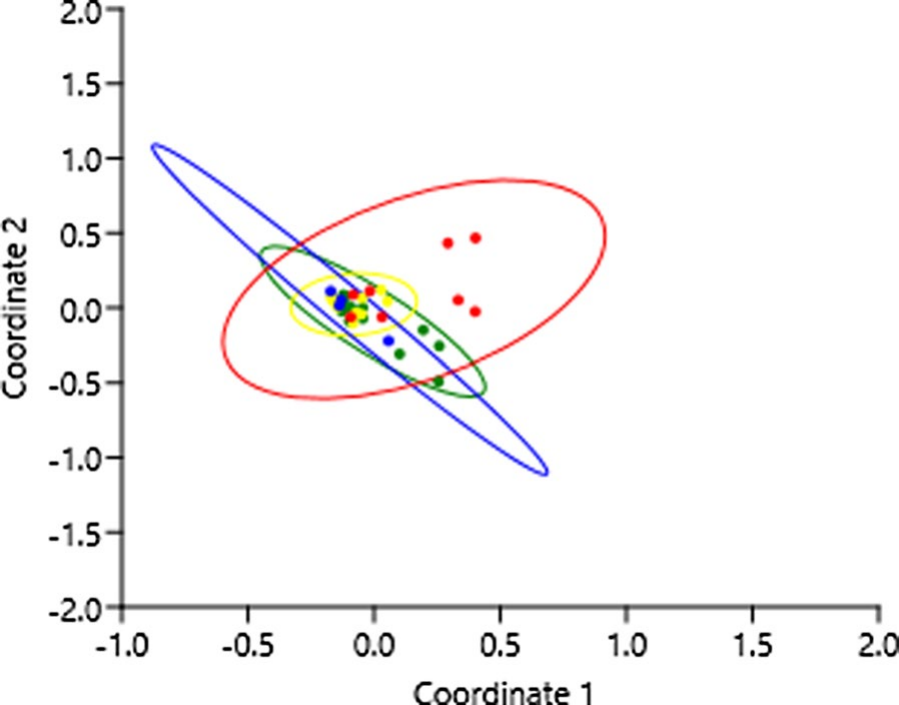
Principal coordinate analysis (PCoA) showing the similarity between the species indicated by the 35 traders in the four mesoregions. Green = Mata; yellow = Agreste; blue = Borborema; red = Sertão

Among the recorded species, 27 (16.1% of the total) were found only in the Mata mesoregion. However, the Sertão showed the highest exclusivity, with 45 species (26.8%). The Agreste mesoregion had only four exclusive species (2.4%), while the Borborema mesoregion had only three exclusive species (1.8%) (Table 1). The one-way ANOSIM multivariate analysis demonstrated similarity among the set of species traded in the mesoregions, with exception for the Sertão, which was significantly different from all other mesoregions (R = 0.2136; *p* < 0.0018), showing significant variation compared to the Mata (R = 0.2632; *p* < 0.0074), Agreste (R = 0.3752; *p* < 0.0036), and Borborema mesoregions (R = 0.3888; *p* < 0.0187). The principal coordinate analysis (PCoA) highlighted the similarity between the plant species traded by the informants in three of the mesoregions compared to the Sertão (Fig. 5).

The four mesoregions shared 35 species. The one-way ANOSIM multivariate analysis demonstrated significant similarity between the mesoregions, except for the Sertão, which showed a significant difference from all remaining mesoregions. The difference between the Sertão and the remaining mesoregions could also be observed in the principal coordinates analysis (Fig. 5).

### Species presence and absence in the trade points

During the study period, 88 species were unavailable at least once at least at one of the traders interviewed (Table 3). The Mata was the mesoregion where the highest number of species was absent at some point during the year, while the Borborema was the region with least seasonal absence of species.

**Table 1:** Medicinal plants traded in public markets of the Mata, Agreste, Borborema, and Sertão mesoregions of Paraíba, northeastern Brazil, indications, and categories of medicinal use and relative importance (RI) of each species

Family Scientific name	Local/Vernacular name	Medicinal use categories	Mesoregion [number of vendors]	RI
**Acanthaceae**				
*Justicia pectoralis* Jacq	Chachambá	RES (cough, hoarseness, expectoration); DIS (indigestion)	Mata [6]	0.47
**Adoxaceae**				
*Sambucus australis* Cham. & Schltdl	Sabugueira	NES (tranquilizer), UDS (fever), BCS (high blood pressure), PAD (measles, infection), RES (cough, flu)	Mata [3]; Agreste [5]; Borborema [3]; Sertão [5]	0.81
**Alismataceae**				
*Echinodorus* sp.	Chapéu de couro	ENM (diabetes, weight loss, uric acid); BCS (immunity); MSS (bone pain, joints); UDS (pain)	Mata [2]; Sertão [3]	0.88
**Amaranthaceae**				
*Dysphania ambrosioides* (L.) Mosyakin & Clemants	Mentruz	RES (flu, expectoration), PAD (worm infection)	Borborema [1]	0.22
*Gomphrena demissa* Mart	Capitãozinho	RES (cough, flu, expectoration)	Sertão [3]	0.3
**Amaryllidaceae**				
*Allium sativum* L	Alho	ENM (cholesterol, "reduce the levels," weight loss); RSS (erectile dysfunction); BCS (anticoagulant, stroke); PAD (worm infection); RES (tiredness)	Mata [1]; Sertão [1]	1.06
*Allium* sp.	Cebolinha branca	RES (cough, flu, hoarseness, expectoration, bronchitis, sinusitis, common cold); UDS (fever); DIS (baby colic); URS (diuretic)	Mata [9]; Agreste [8]; Borborema [3]; Sertão [4]	1.07
**Anacardiaceae**				
*Anacardium occidentale* L	Cajú roxo	SST (wound healing); UDS (inflammation); DIS (intestine inflammation); EXI (stop bleeding); PAD (antibiotic); RSS (uterus inflammation, vaginal discharge, prostate)	Mata [7]; Agreste [5]; Borborema [1]; Sertão [7]	1.17
*Myracrodruon urundeuva* Allemão	Aroeira	UDS (inflammation); URS (prostate); SST (wound healing, pruritus); DIS (gastritis); EXI (stop bleeding); MSS (bone inflammation); PAD (antibiotic); ENM (menopause); BCS (high blood pressure, blood)	Mata [2]; Agreste [4]; Sertão [6]	1.69
*Schinus terebinthifolia* Raddi	Aroeira	UDS (inflammation); SST (wound healing); DIS (intestine inflammation)	Mata [4]	0.52
*Schnopsis brasiliensis* Engl	Baraúna	UDS (inflammation); SST (wound healing); URS (kidneys); MSS (spine); RSS (prostate)	Mata [1]; Agreste [1]; Sertão [3]	0.87
**Annonaceae**				
*Annona muricata* L	Graviola	DIS (gastritis); NEO (cancer); ENM ("reduce the levels” diabetes); UDS (inflammation)	Mata [2]; Sertão [3]	0.76
*Xylopia aromática* (Lam.) Mart	Imbira	DIS (bellyache, indigestion, stomach, stomach ache); UDS (pain); RSS (menstrual cramps); MSS (back pain)	Mata [3]; Agreste [1]; Sertão [4]	0.88
**Apiaceae**				
*Anethum graveolens* L	Endro	NES (tranquilizer, insomnia); UDS (pain); BCS (heart, jaundice, high blood pressure, tachycardia); DIS (intestinal colic, stomach ache, gallbladder pain, constipation, indigestion); RSS (menstrual cramps)	Mata [4]; Agreste [1]; Borborema [3]; Sertão [8]	1.37
*Centella* sp.	Centelha asiática	ENM (weight loss)	Mata [1]; Sertão [1]	0.17
*Coriandrum sativum* L	Coentro	BCS (high blood pressure); ENM (cholesterol, menopause); DIS (indigestion, throat problems); UDS (dizziness, headache); SES (labyrinthitis)	Mata [1]; Agreste [1]; Borborema [1]; Sertão [3]	1.06
*Foeniculum vulgare* Mill	Erva doce	NES (tranquilizer, depression, insomnia); RES (sinusitis, runny nose); BCS (high blood pressure, heart); UDS (fever, dizziness, headache); INT (Intoxication); ENM (menopause); DIS (bellyache)	Mata [13]; Agreste [10]; Borborema [4]; Sertão [8]	1.59
*Pimpinella anisum* L	Erva doce	NES (tranquilizer, depression, insomnia); RES (sinusitis, runny nose); BCS (high blood pressure, heart); UDS (fever, dizziness, headache); INT (Intoxication); ENM (menopause); DIS (bellyache)	Mata [13]; Agreste [10]; Borborema [4]; Sertão [7]	1.59
**Apocynaceae**				
*Hancornia speciosa* Gomes	Mangaba	BCS (high blood pressure)	Mata [1]	0.17
**Aquifoliaceae**				
*Ilex* sp.	Chá mate	DIS (indigestion)	Mata [2]; Borborema [1]	0.17
**Arecaceae**				
*Cocos nucifera* L	Côco	BCS (jaundice); URS (diuretic)	Sertão [1]	0.35
*Copernicia prunifera* (Mill.) H.E. Moore	Carnaúba	URS (kidneys)	Sertão [2]	0.17
*Syagrus oleracea* (Mart.) Becc	Côco catolé	MSS (spine); URS (kidneys, kidney stones, urinary tract infection); DIS (gallstones)	Mata [1]; Agreste [3]; Sertão [5]	0.65
**Aristolochiaceae**				
*Aristolochia* sp.	Cipó-de-mil-homem	DIS (indigestion)	Mata [1]	0.17
**Asparagaceae**				
*Agave* sp.	Agave branco	UDS (inflammation); ENM (cholesterol)	Agreste [1]; Sertão [1]	0.35
*Sansevieria trifasciata* Prain	Espada de São Jorge	URS (kidneys); UDS (inflammation)	Sertão [1]	0.35
**Asphodelaceae**				
*Aloe vera* (L.) Burm. f	Babosa	SST (wound healing)	Mata [1]	0.17
**Asteraceae**				
*Acanthospermum hispidum* DC	Espinho de cigano	RES (flu, bronchitis)	Sertão [1]	0.24
*Ageratum conyzoides* L	Mentraste	RSS (regulation of menstruation); UDS (inflammation)	Sertão [2]	0.35
*Artemisia* sp.	Artemísia	UDS (inflammation)	Mata [1]	0.17
*Baccharis* sp.	Carqueja	ENM (liver, liver fat, weight loss, diabetes, cholesterol, blood fat); URS (diuretic, kidneys)	Mata [8]; Agreste [1]; Borborema 1]; Sertão [4]	0.72
*Bidens pilosa* L	Picão preto	UDS (inflammation)	Mata [1]	0.17
*Egletes viscosa* (L.) Less	Macela	DIS (indigestion, diarrhea, stomach, liver, intestine, intestinal infection); ENM (diabetes)	Mata [9]; Agreste [3]; Borborema [1]; Sertão [7]	0.66
*Gymnanthemum amygdalinum* (Delile) Sch. Bip. ex Walp	Alcachofra	ENM (cholesterol, diabetes, liver inflammation, liver fat)	Mata [3]; Borborema [1]; Sertão [4]	0.36
*Helianthus annuus* L	Girassol	UDS (dizziness); SES (labyrinthitis); BCS (thrombosis, CVA); musculoskeletal system (rheumatism, bursitis); BCS (high blood pressure)	Mata [6]; Agreste [7]; Borborema [1]; Sertão [5]	0.99
*Matricaria chamomilla* L	Camomila	NES (tranquilizer, depression, insomnia); UDS (fever); NEO (cancer); URS (urethral inflammation); SST (lighten skin spots); BCS (high blood pressure)	Mata [12]; Agreste [9]; Borborema [3]; Sertão [7]	1.17
*Solidago chilensis* Meyen	Arnica	UDS (pain); MSS (joints); EXI (wounds, blood clot)	Mata [1]; Sertão [3]	0.58
*Taraxacum officinale* F.H. Wigg	Dente de leão	ENM (liver fat, weight loss); NES (memory); MSS (joints); DIS (intestine); BCS (blood circulation)	Mata [2]; Borborema [1]; Sertão [2]	0.93
*Vernonanthura phosphorica* (Vell.) H. Rob	Assa peixe	URS (kidneys)	Mata [1]	0.17
**Bignoniaceae**				
*Anemopaegma* sp.	Catuaba	UDS (inflammation); RSS (aphrodisiac); MSS (bone pain)	Mata [2]; Sertão [2]	0.52
*Handroanthus heptaphyllus* (Vell.) Mattos	Pau d'arco roxo	UDS (pain, inflammation); NEO (cancer); RSS (cysts, myoma, ovary problems); MSS (rheumatism)	Mata [3]; Sertão [2]	0.88
*Handroanthus* sp.	Pau d'arco	URS (kidneys)	Agreste [1]	0.17
*Jacaranda* sp.	Caroba	SST (pruritus, skin irritation); BCS (depurative)	Sertão [2]	0.41
**Bixaceae**				
*Bixa orellana* L	Urucum	ENM (reduce cholesterol)	Mata [2]; Sertão [1]	0.17
**Boraginaceae**				
*Heliotropium indicum* L	Fedegoso	PRP ("female cleaning after childbirth")	Sertão [2]	0.17
*Heliotropium nicotianaefolium* Poir	Sete Sangrias	URS (kidneys)	Mata [1]; Sertão [4]	0.17
*Symphytum officinale* L	Confrei	ENM (uric acid); SES (labyrinthitis); UDS (inflammation)	Mata [2]; Sertão [1]	0.52
*Varronia curassavica* Jacq	Erva balieira	UDS (inflammation)	Mata [1]	0.17
**Brassicaceae**				
*Brassica* sp.	Mostarda	BCS (thrombosis, CVA, stroke, circulation); MSS (rheumatism); UDS (pain, headache, swelling)	Mata [6]; Agreste [4]; Borborema [1]; Sertão [2]	0.83
*Rorippa nasturtium-aquaticum* (L.) Hayek	Agrião	RES (bronchitis, asthma)	Sertão [1]	0.24
**Burseraceae**				
*Commiphora leptophloeos* (Mart.) J.B. Gillett	Imburana	RES (flu), MSS (osteoarthritis)	Mata [1]; Agreste [1]; Sertão [2]	0.35
**Cactaceae**				
*Cereus jamacaru* DC	Cardeiro	URS (kidneys)	Sertão [2]	0.17
*Melocactus zehntneri* (Britton & Rose) Luetzelb	Coroa de Frade	RES (tiredness, asthma)	Mata [1]	0.24
**Capparaceae**				
*Cynophalla flexuosa* (L.) J. Presl	Feijão brabo	MSS (back pain)	Sertão [1]	0.17
**Caprifoliaceae**				
*Valeriana* sp.	Valeriana	NES (tranquilizer)	Mata [1]	0.17
**Caryocaraceae**				
*Caryocar* sp.	Pequi	RES (flu, cough)	Sertão [1]	0.24
**Celastraceae**				
*Monteverdia rigida* (Mart.) Biral	Bom nome	UDS (pain, inflammation, infection), MSS (fracture healing, joints); EXI (bumps); URS (kidneys, urinary tract infection)	Mata [1]; Sertão [2]; Agreste [4]	0.94
*Monteverdia ilicifolia* (Mart. ex Reissek) Biral	Espinheira santa	DIS (stomach, stomach pain, gastritis, ulcer, gastroesophageal reflux, heartburn, hepatitis, cirrhosis); MSS (joint pain); UDS (inflammation); BCS (anticoagulant)	Mata [8]; Agreste [7]; Borborema [1]; Sertão [5]	1.13
**Chrysobalanaceae**				
*Microdesmia rigida* (Benth.) Sothers & Prance	Oiticica	ENM (diabetes), MSS (muscle strain)	Mata [5]; Sertão [3]	0.35
**Cleomaceae**				
*Cleome spinosa* Jacq	Mussambê	RES (expectoration, cough)	Sertão [1]	0.24
**Combretaceae**				
*Combretum fruticosum* (Loefl.) Stuntz	Mufumbo	RES (flu, cough)	Sertão [1]	0.24
*Combretum glaucocarpum* Mart	João Mole	BCS (swollen heart)	Mata [1]	0.17
**Convolvulaceae**				
*Operculina macrocarpa* (L.) Urb	Batata de purga	DIS (purgative), BCS (anticoagulant, hemorrhoids), UDS (inflammation)	Mata [1]; Agreste [3]; Sertão [2]	0.58
**Costaceae**				
*Costus spicatus* (Jacq.) Sw	Cana da índia	URS (kidney problems, urinary tract infection)	Mata [1]	0.24
**Cucurbitaceae**				
*Luffa operculata* (L.) Cogn	Cabacinha	RES (sinusitis); SST (shrink lumps)	Mata [3]; Agreste [2]; Sertão [3]	0.35
*Momordica charantia* L	Melão de São Caetano	BCS (hemorrhoids)	Sertão [1]	0.17
*Sicana odorifera* (Vell.) Naudin	Croá	RSS (cysts, myoma), ENM (thyroid), URS (kidney stones), UDS (inflammation)	Mata [1]; Agreste [1]	0.76
*Wilbrandia* sp.	Cabeça de negro	ENM (blood clotting), SST (shrink lumps)	Mata [1]; Agreste [1]; Sertão [2]	0.35
**Erythroxylaceae**				
*Erythroxylum* sp.	Rompe gibão	MSS (back pain, joints), NES (nerves)	Sertão [3]	0.41
**Equisetaceae**				
*Equisetum giganteum* L	Cavalinha	URS (kidney stones, urinary tract infection, kidneys, diuretic), ENM (weight loss); UDS (infection); NEO (breast cysts); RSS (prostate); UDS (inflammation)	Mata [9]; Sertão [5]	1.17
**Euphorbiaceae**				
*Cnidoscolus quercifolius* Pohl	Favela	UDS (inflammation); URS (kidney stones); DIS (gastritis, ulcer); RSS (vaginal discharge, myoma, cysts, prostate)	Agreste [3]; Sertão [3]	0.94
*Cnidoscolus urens* (L.) Arthur	Urtiga branca	URS (urinary tract infection, urine cleaning); MSS (spine inflammation); UDS (inflammation, infection); SST (wound healing); DIS (appendix); RSS (prostate); NEO (cancer); RES (cough)	Mata [6]; Agreste [2]; Sertão [2]	1.51
*Croton* sp.	Velame branco	MSS (bone pain, rheumatism);	Agreste [2]; Sertão [2]	0.24
**Fabaceae**				
*Abarema cochliacarpos* (Gomes) Barneby & J.W. Grimes	Babatenom	UDS (inflammation); SST (wound healing); NEO (cancer); PAD (antibiotic); RSS (uterus inflammation); MSS (bone inflammation); DIS (gastritis)	Mata [6]; Agreste [5]; Borborema [1]; Sertão [6]	1.22
*Amburana cearensis* (Allemão) A.C. Smith	Cumarú	RES (expectorant, sinusitis, flu, cough); BCS (hemorrhoid); UDS (inflammation)	Mata [5]; Agreste [3]; Borborema [1]; Sertão [6]	0.71
*Anadenanthera colubrina* (Vell.) Brenan	Angico	RES (cough, flu, expectorant); UDS (inflammation)	Mata [3]; Agreste [3]; Sertão [3]	0.47
*Bauhinia* sp.^1^	Mororó	ENM (diabetes, cholesterol)	Mata [3]; Sertão [5]	0.24
*Bauhinia* sp.^**2**^	Pata de vaca	ENM (diabetes, cholesterol); MSS (spine problems); UDS (inflammation)	Mata [2]; Agreste [1]; Borborema [1]; Sertão [2]	0.58
*Cajanus cajan* (L.) Huth	Feijão gandú	NEO (intestinal cancer), ENM (diabetes), BCS (thrombosis)	Mata [1]	0.52
*Cenostigma pyramidale* (Tul.) E. Gagnon & G.P. Lewis	Catingueira	RES (cough, flu); DIS (bellyache); ENM (cholesterol)	Agreste [1]; Sertão [4]	0.58
*Erythrina velutina* Willd	Mulungú	NES (insomnia, tranquilizer, nerve weakness, memory); RES (cough)	Mata [4]; Agreste [2]; Sertão [5]	0.53
*Glycyrrhiza glabra* L	Alcaçuz	RES (expectoration)	Mata [1]	0.17
*Hymenaea courbaril* L	Jatobá	RES (expectoration, cough, flu); RSS (prostate inflammation; cysts; erectile dysfunction); URS (kidney problems); BCS (anemia); ENM (fortifying, rickets) UDS (inflammation, pain)	Mata [5]; Agreste [3]; Borborema [1]; Sertão [5]	1.42
*Libidibia ferrea* (Mart. ex Tul.) L.P. Queiroz	Jucá	MSS (bone pain, spine, tendinitis, bursitis, spine inflammation); URS (kidney pain); SST (lumps); RSS (cysts); UDS (inflammation); RES (expectorant)	Mata [1]; Agreste [1]; Sertão [5]	1.29
*Mimosa tenuiflora* (Willd.) Poir	Jurema preta	UDS (inflammation); SST (wound healing); BCS (hemorrhoid)	Mata [2]; Sertão [3]	0.52
*Mimosa* sp.	Malícia	SST (wound healing)	Sertão [1]	0.17
*Mucuna urens* (L.) Medik	Coronha	UDS (inflammation, pain); MSS (herniated disc, spine inflammation)	Agreste [1]; Sertão [2]	0.65
*Myroxylon peruiferum* L. f	Bálsamo	RES (expectorant)	Mata [1]; Sertão [1]	0.17
*Piptadenia* sp.	Jurema branca	DIS (gastritis)	Sertão [2]	0.17
*Pterodon emarginatus* (Vogel.) Kunth	Sucupira	MSS (spine pain, spine inflammation, herniated disc, joint pain, bone pain, bone inflammation, osteoarthritis), DIS (sore throat); ENM (diabetes); BCS (thrombosis; high blood pressure); RES (sinusitis, tonsillitis); URS (kidneys)	Mata [7]; Agreste [4]; Borborema [1]; Sertão [5]	1.54
*Senna* sp.	Sena	DIS (indigestion; constipation, "release dry feces," intestinal colic); ENM (weight loss); RES (cough); PAD (worm infection); UDS (fever, infection)	Mata [6]; Agreste [2]; Borborema [1]; Sertão [7]	1.12
*Tamarindus indica* L	Tamarindo	BCS (anemia)	Mata [2]; Sertão [2]	0.17
**Humiriaceae**				
*Endopleura uchi* (Huber) Cuatrec	Uxi amarelo	RSS (uterus, cysts, polycysts, myoma, uterus inflammation)	Mata [1]; Sertão [4]	0.42
**Illiaceae**				
*Illicium verum* Hooker	Anil estrelado	BCS (heart, high blood pressure); MSS (bone pain, back pain); DIS (indigestion, bellyache, liver, stomach ache); UDS (pain); RSS (colic)	Mata [4]; Agreste [6]; Borborema [3]; Sertão [3]	1.18
**Lamiaceae**				
*Lavandula angustifolia* Mill	Alfazema	DIS (baby colic, constipation, intestine); RES (cough); NES (tranquilizer), UDS (colic, pain, fever), CUD (“evil eye”), BCS (jaundice); PAD (infection)	Mata [7]; Agreste [3]; Borborema [2]; Sertão [4]	1.4
*Melissa officinalis* L	Melissa	NES (tranquilizer)	Mata [1]	0.17
*Mentha* sp.	Hortelã	RES (flu, expectoration); ENM (weight loss); PAD (“bacteria in the stomach”); BCS (prevent strokes)	Mata [1]; Sertão [2]	0.76
*Mesosphaerum suaveolens* (L.) Kuntze	Alfazema braba	DIS (bellyache, constipation, intestine); ENM (cholesterol, diabetes, weight loss)	Sertão [1]	0.6
*Ocimum* sp.^1^	Manjericão	CUD ("bad air"); RES (flu)	Mata [2]; Sertão [1]	0.35
*Ocimum* sp.^2^	Alfavaca	RES (sinusitis)	Mata [2]	0.17
*Origanum* sp.	Orégano	ENM (menopause); NEO (cancer); DIS (constipation); NES (insomnia); PAD (candidiasis); RES (cough)	Mata [2]; Agreste [1]; Sertão [1]	1.04
*Rosmarinus officinalis* L	Alecrim	BCS (arrhythmia, high blood pressure, heart problems, circulation, CVA); DIS (stomach ache, constipation, indigestion); NES (depression, tranquilizer, meningitis); UDS (headache); RES (sinusitis, tiredness, asthma); ENM (thyroid)	Mata [10]; Agreste [6]; Borborema [3]; Sertão [4]	1.67
*Salvia hispanica* L	Chia	ENM (weight loss, appetite suppressant, menopause); DIS (intestinal regulation)	Mata [3]; Agreste [1]; Borborema [1]; Sertão [3]	0.47
*Salvia officinalis* L	Sálvia	ENM (weight loss)	Mata [1]	0.17
*Vitex gardneriana* Schauer	Jaramataia	URS (kidneys, prostate), ENM (diabetes, cholesterol)	Sertão [4]	0.47
**Lauraceae**				
*Cinnamomum* sp.	Canela	NES (tranquilizer, stimulant); ENM (weight loss, diabetes); BCS (low blood pressure, prevent blood clotting); DIS (throat, stomach ache); RES (hoarseness); UDS (vomiting); MSS (bones); EXI (bumps)	Mata [7]; Agreste [6]; Borborema [3]; Sertão [8]	1.64
*Laurus nobilis* L	Louro	DIS (diarrhea, indigestion, stomach); UDS (headache); URS (kidneys); UDS (headache)	Mata [6]; Agreste [4]; Borborema [2]; Sertão [3]	0.82
*Persea americana* Mill	Abacate	BCS (heart); URS (kidneys)	Agreste [1]; Borborema [2]; Sertão [2]	0.35
**Lecythidaceae**				
*Bertholletia excelsa* Bonpl	Castanha do Pará	ENM (liver fat)	Mata [1]; Sertão [1]	0.17
**Linaceae**				
*Linum usitatissimum* L	Linhaça	DIS (constipation, intestinal regulation); BCS (thrombosis, CVA); MSS (rheumatism); ENM (weight loss)	Mata [2]; Agreste[2]; Sertão [2]	0.82
**Lyrthaceae**				
*Punica granatum* L	Romã	DIS (sore throat, gastritis, stomach, heartburn); RES (hoarseness, cough; tonsillitis); RSS (prostate, erectile dysfunction); BCS (heart); ENM (liver fat); UDS (inflammation); PAD (antibiotic); SST (wound healing); URS (kidneys)	Mata [7]; Agreste [4]; Borborema [1]; Sertão [3]	2
**Malvaceae**				
*Abelmoschus esculentus* (L.) Moench	Quiabo	BCS (CVA); MSS (bones)	Sertão [2]	0.35
*Chorisia glaziovii* (Kuntze) E. Santos	Barriguda	MSS (spine); URS (kidneys)	Sertão [3]	0.35
*Gossypium herbaceum* L	Algodão	SST (furuncles)	Sertão [1]	0.17
*Hibiscus* sp.	Hibisco	ENM (diabetes, cholesterol, triglycerides, liver fat, weight loss); URS (diuretic); BCS (blood circulation, swelling, low immunity, blood pressure, prevent blood clotting); UDS (inflammation); DIS (liver inflammation)	Mata [10]; Agreste [2]; Borborema [1]; Sertão [8]	1.37
*Pseudobombax marginatum* (A. St.-Hil., Juss. & Cambess.) A. Robyns	Imbiratanha	MSS (spine); URS (kidneys, kidney inflammation)	Sertão [5]	0.41
**Melastomataceae**				
*Miconia albicans* (Sw.) Triana	Canela de velho	MSS (arthritis, osteoarthritis, bursitis, herniated disc, bone pain, joint pain, tendinitis, rheumatism, bone inflammation); UDS (pain, infection, headache, inflammation); PAD (Chikungunya)	Mata [10]; Agreste [3]; Borborema [2]; Sertão [4]	1.21
**Meliaceae**				
*Cedrela odorata* L	Cedro	DIS (intestinal problems, constipation, bellyache); UDS (pain, inflammation)	Mata [2]; Sertão [1]	0.53
**Menispermaceae**				
*Cissampelos sympodialis* Eichler	Milona	RES (cough, flu); ENM (diabetes, liver fat)	Sertão [3]	0.47
**Monimiaceae**				
*Peumus boldus* Molina	Boldo do Chile	DIS (diarrhea, liver problems, stomach ache, indigestion, intestine, stomach problems, sulfur burps, gastritis); URS (kidneys); MBD (hangover); ENM (liver fat)	Mata [13]; Agreste [9]; Borborema [3]; Sertão [8]	1.13
**Moraceae**				
*Brosimum gaudichaudii* Trécul	Mamica de cadela	SST (Vitiligo)	Mata [1]	0.17
*Morus* sp.	Amora	ENM (diabetes, menopause, weight loss, cholesterol); URS (diuretic); BCS (boost immunity, high blood pressure); UDS (inflammation)	Mata [7]; Borborema [1]; Sertão [4]	0.94
**Moringaceae**				
*Moringa* sp.	Moringa	NES (memory); NEO (cancer)	Sertão [1]	0.35
**Musaceae**				
*Musa* x *paradisiaca* L	Banana	RES (cough)	Sertão [1]	0.17
**Myristicaceae**				
*Myristica fragans* Houtt	Noz moscada	SES (labyrinthitis); BCS (prevent strokes)	Mata [1]; Agreste [1]; Sertão [1]	0.35
**Myrtaceae**				
*Eucalyptus globulus* Labill	Eucalipto	RES (sinusitis, common cold, flu, expectorant); NES (tranquilizer); DIS (bellyache); UDS (fever); MSS (bone pain)	Mata [4]; Agreste [4]; Borborema [3]; Sertão [5]	1.06
*Eugenia uniflora* L	Pitanga	DIS (bellyache)	Sertão [1]	0.17
*Myrcia speciosa* (Amshoff) McVaugh	Pedra-hume-kar	ENM (diabetes)	Mata [1]	0.17
*Psidium guajava* L	Guava	DIS (bellyache)	Sertão [1]	0.17
*Syzygium aromaticum* (L.) Merr. & L. M. Perry	Cravo	UDS (headache, dizziness, bad breath); DIS (indigestion; toothache); SES (Labyrinthitis); NES (Insomnia, tranquilizer); BCS (high blood pressure)	Mata [4]; Agreste [3]; Borborema [3]; Sertão [3]	1.18
*Syzygium cumini* (L.) Skeels	Oliveira	ENM (weight loss, "reduce the levels"); NEO (cancer)	Sertão [2]	0.41
**Nyctaginaceae**				
*Boerhavia diffusa* L	Pega pinto	URS (kidneys, urinary tract infection)	Sertão [1]	0.24
**Olacaceae**				
*Ximenia americana* L	Ameixa	DIS (gastritis); UDS (inflammation); SST (wound healing); ENM (cholesterol)	Mata [1]; Agreste [2]; Borborema [1]; Sertão [6]	0.69
**Opiliaceae**				
*Agonandra brasiliensis* Miers ex Benth. & Hook. f	Marfim	RES (cough, flu)	Mata [1]	0.24
**Papaveraceea**				
*Argemone mexicana* L	Cardo santo	BCS (CVA, thrombosis)	Mata [3]; Agreste [1]; Sertão [1]	0.24
**Passifloraceae**				
*Turnera subulata* Smith	Chanana	URS (urinary tract infection)	Sertão [2]	0.17
**Pedaliaceae**				
*Sesamum orientale* L	Gergelim preto	BCS (thrombosis, CVA); MSS (bone pain, joint pain, rheumatism, bone calcium); UDS (numbness); DIS (intestinal regulation); ENM (menopause, appetite suppressant, weight loss)	Mata [5]; Agreste [5]; Sertão [3]	1.24
**Petiveriaceae**				
*Petiveria alliacea* L	Tipi	MSS (rheumatism)	Mata [1]; Sertão [1]	0.17
**Phyllanthaceae**				
*Phyllanthus niruri* L	Quebra pedra	URS (kidney stones, kidney problems); DIS (liver, gallstones); EXI (bumps)	Mata [2]; Borborema [1]; Sertão [2]	0.65
**Piperaceae**				
*Piper nigrum* L	Pimenta do reino	NES (labyrinthitis); UDS (headache)	Mata [2]; Borborema [1]; Sertão [1]	0.35
**Poaceae**				
*Cymbopogon citratus* (DC.) Stapf	Capim santo	NES (tranquilizer, stimulant); DIS (bellyache); BCS (high blood pressure)	Mata [2]; Agreste [1]; Sertão [2]	0.58
*Zea mays* L	Milho	URS (kidneys), BCS (jaundice)	Sertão [1]	0.35
**Polygonaceae**				
*Polygonum hydropiperoides* Michx	Erva de bicho	URS (kidneys)	Mata [1]	0.17
**Rhamnaceae**				
*Ziziphus joazeiro* Mart	Juá	SST (dandruff); DIS (gum disease)	Mata [2]; Sertão [3]	0.35
**Rubiaceae**				
*Coutarea hexandra* (Jacq.) K. Schum	Quina quina	ENM (diabetes, blood clotting), RES (sinusitis), MSS (rheumatism), UDS (fever)	Mata [3]; Agreste [1]; Sertão [5]	0.76
*Genipa americana* L	Jenipapo	MSS (fracture healing); BCS (increase blood platelets)	Sertão [3]	0.35
*Guettarda* sp.	Angélica	RSS (menstrual cramps)	Sertão [1]	0.17
*Morinda citrifolia* L	Noni	ENM (diabetes, weight loss); NEO (cancer); BCS (blood circulation; fluid retention, hemorrhoids, high blood pressure); DIS (gastritis); RSS (uterus inflammation)	Agreste [1]; Sertão [1]	1.12
*Uncaria* sp.	Unha de gato	UDS (inflammation); RSS (cysts, nodule, myoma, polycysts, uterus inflammation); MSS (bone inflammation)	Mata [4]; Agreste [2]; Sertão [4]	0.77
**Rutaceae**				
*Citrus aurantium* L	Laranja	NES (tranquilizer)	Mata [1]; Sertão [2]	0.17
*Pilocarpus* sp.	Jaborandi	UDS (fever)	Mata [1]	0.17
**Sapotaceae**				
*Sideroxylon obtusifolium* (Roem & Schult.) T.D. Penn	Quixaba	UDS (inflammation, pain); MSS (spine inflammation); SST (wound healing); URS (kidneys); EXI (bumps)	Mata [5]; Agreste [6]; Borborema [1]; Sertão [5]	0.93
**Selaginellaceae**				
*Selaginella convoluta* (Arn.) Spring	Mão fechada	RES (cough, flu)	Sertão [1]	0.24
**Smilacaceae**				
*Smilax* sp.	Japecanga	SST (vitiligo); MSS (spine inflammation)	Mata [1]	0.35
**Solanaceae**				
*Solanum americanum* Mill	Erva moura	SST (wound healing)	Sertão [1]	0.17
*Solanum paniculatum* L	Jurubeba	DIS (liver, gastritis); ENM (diabetes); RSS (menstrual cramps)	Sertão [2]	0.58
**Theaceae**				
*Camellia sinensis* (L.) Kuntze	Chá verde/Chá preto	ENM (cholesterol, diuretic, weight loss, loss of appetite); NES (nervousness); UDS (inflammation, fever); DIS (bellyache, intestine, intestinal infection); SES (vision problems); URS (diuretic)	Mata [9]; Agreste [4]; Borborema [3]; Sertão [6]	1.42
**Verbenaceae**				
*Lippia alba* (Mill.) N.E. Br. ex Britton & P. Wilson	Erva cidreira	RES (expectoration); BCS (anemia); DIS (indigestion, bellyache); NES (tranquilizer, insomnia); ENM (whet the appetite)	Mata [8]; Agreste [1]; Sertão [5]	0.99
**Violaceae**				
*Pombalia lanata* (A. St.-Hil.) Paula-Souza	Papaconha	RES (expectoration, cough, flu); PAD (worm infection); UDS (fever)	Mata [1]; Borborema [1]; Sertão [6]	0.65
**Vitaceae**				
*Cissus* sp.	Parreira	URS (kidneys); MSS (spine)	Sertão [3]	0.35
**Zingiberaceae**				
*Alpinia zerumbet* (Pers.) B.L. Burtt & R.M. Sm	Colônia	UDS (fever); RES (expectoration)	Mata [1]; Sertão [2]	0.35
*Curcuma longa* L	Cúrcuma	UDS (inflammation); BCS (hepatitis; jaundice); PAD (antibiotic); RES (flu); MSS (bones)	Mata [4]; Sertão [1]	0.93
*Zingiber officinale* Roscoe	Gengibre	BCS (high blood pressure, prevent blood clotting); DIS (throat pain, sore throat, stomach); RES (cough, expectoration, flu, hoarseness); ENM (liver fat, cholesterol, weight loss); UDS (pain, inflammation); NES (stimulant); URS (diuretic)	Mata [9]; Agreste [7]; Borborema [3]; Sertão [4]	1.78
**Indeterminated**				
Indeterminated 1	Baço	RES (cough, flu, tiredness), UDS (pain)	Mata [1]; Borborema [1]; Sertão [1]	0.47
Indeterminated 2	Caninana	SST (wound healing)	Sertão [1]	0.17
Indeterminated 3	Catinga branca	DIS (diarrhea)	Borborema [1]	0.17
Indeterminated 4	Cauaçú	DIS (gastritis, ulcer)	Borborema [1]	0.24
Indeterminated 5	Chocalho de vaqueiro	MSS (bone pain, joint pain, rheumatism)	Sertão [4]	0.3
Indeterminated 6	Cipó de cruz	MSS (joints, rheumatism, spine), UDS (inflammation)	Sertão [2]	0.47
Indeterminated 7	Espriteira	RES (common cold, flu)	Agreste [1]	0.24
Indeterminated 8	Jalapa	ENM (diabetes)	Mata [1]	
Indeterminated 9	Junço	UDS (pain)	Sertão [1]	0.17
Indeterminated 10	Mapirunga	MSS (rheumatism), UDS (pain)	Agreste [1]	0.28
Indeterminated 11	Maria leite	URS (kidney stones), DIS (gallstone)	Mata [1]	0.34
Indeterminated 12	Pau tenente	ENM (diabetes, cholesterol), UDS (inflammation)	Mata [1]; Sertão [3]	0.41
Indeterminated 13	Pimenta parda	RES (throat)	Agreste [1]	0.17
Indeterminated 14	Porangaba	ENM (cholesterol, blood clotting, diabetes), DIS (intestine)	Mata [2]; Agreste [1]; Sertão [4]	0.47
Indeterminated 15	Quebra faca	ENM (diabetes, cholesterol), SST (pruritus)	Sertão [2]	0.41
Indeterminated 16	Sassafrás	URS (urinary tract infection, kidneys)	Sertão [2]	0.24
Indeterminated 17	Urinana	URS (urinary tract infection), ENM (retained fat)	Mata [1]	0.35

Categories: BCS = blood and cardiovascular system; CUD = cultural diseases; DIS = digestive system; ENM = endocrine system, nutrition, and metabolism; EXI = external injuries; INT = intoxication; MBD = mental and behavioral diseases; MSS = musculoskeletal system; NEO = neoplasms; NES = nervous system; PAD = parasitic diseases; PRP = pregnancy and parturition; RES = respiratory system; RSS = reproductive system and sexual health; SES = sensory system; SST = skin and subcutaneous tissue; UDS = unspecified diseases and symptoms; URS = urinary system

**Table 2:** Medicinal plants of high relative importance in public markets of different mesoregions in the state of Paraíba, northeastern Brazil

Species	Origin	RI	Species	Origin	RI
*Punica granatum*	E	2	*Abarema cochliacarpos*	N	1.22
*Zingiber officinale*	E	1.78	*Miconia albicans*	N	1.21
*Myracrodruon urundeuva*	N	1.69	*Illicium verum*	E	1.18
*Rosmarinus officinalis*	E	1.67	*Syzygium aromaticum*	E	1.18
*Cinnamomum* sp.	E	1.64	*Anacardium occidentale*	N	1.17
*Foeniculum vulgare*	E	1.59	*Matricaria chamomilla*	E	1.17
*Pimpinella anisum*	E	1.59	*Equisetum giganteum*	N	1.17
*Pterodon emarginatus*	N	1.54	*Monteverdia ilicifolia*	N	1.13
*Cnidoscolus urens*	N	1.51	*Peumus boldus*	E	1.13
*Hymenaea courbaril*	N	1.42	*Senna* sp.	N	1.12
*Camellia sinensis*	E	1.42	*Morinda citrifolia*	E	1.12
*Lavandula angustifolia*	E	1.4	*Allium* sp.	E	1.07
*Anethum graveolens*	E	1.37	*Allium sativum*	E	1.06
*Hibiscus* sp.	E	1.37	*Coriandrum sativum*	E	1.06
*Libidibia ferrea*	N	1.29	*Eucalyptus globulus*	E	1.06
*Sesamum orientale*	E	1.24	*Origanum* sp.	E	1.04

N = species native to Brazil. E = exotic species. RI = relative importance

**Table 3:** Percentage of informants that reported the absence of some species during the interview period for each studied mesoregion. Blank cells indicate that the species was not recorded in the mesoregion at any time of the year. 1° = August–October 2017; 2° = November 2017–January 2018; 3° = February–April 2018; 4° = May–July 2018

Species	Mata (N = 13)				Agreste (N = 10)				Borborema (N = 4)				Sertão (N = 8)			
	1°	2°	3°	4°	1°	2°	3°	4°	1°	2°	3°	4°	1°	2°	3°	4°
*Abarema cochliacarpos*	7.69	–	–	–	30	20	20	10	–	–	–	–	–	12.5	–	–
*Agave* sp.					–	–	–	10					–	–	–	–
*Ageratum conyzoides*													–	12.5	12.5	12.5
*Allium* sp.	7.69	–	15.38	7.69	–	30	10	10	–	–	25	–	–	25	12.5	25
*Aloe vera*	7.69	–	15.38	7.69					25	–	25	–				
*Alpinia zerumbet*	15.38	7.69	–	–									–	12.5	–	–
*Amburana cearensis*	15.38	–	7.69	15.38	10	–	30	–	–	–	–	–	–	–	–	–
*Anacardium occidentale*	–	–	7.69	–	–	–	10	10	–	–	–	–	–	12.5	12.5	–
*Anadenanthera colubrina*	15.38	–	–	7.69	–	10	–	–					–	–	–	–
*Anemopaegma* sp.	7.69	7.69	–	–									–	–	12.5	–
*Anethum graveolens*	23.07	–	–	–	–	–	20	20	–	–	–	–	–	12.5	12.5	–
*Annona muricata*	7.69	–	15.38	–	–	–	–	–	–	–	–	–	–	–	–	–
*Baccharis* sp.	15.38	7.69	15.38	–	–	–	–	–	–	–	50	–	25	–	12.5	–
*Bahuinia* sp.^**2**^	7.69	7.69	–	–	10	–	10	–	–	–	–	–	–	–	–	–
*Bertholletia excelsa*	–	–	7.69	–									–	–	–	–
*Boerhavia coccinea*	–	–	7.69	–									–	–	12.5	12.5
*Brassica* sp.	–	–	–	–	10	10	–	–	–	–	–	–	–	–	12.5	12.5
*Camellia sinensis*	7.69	–	–	–	10	20	–	–	–	–	25	25	25	12.5	–	12.5
*Caryocar* sp.													–	–	12.5	12.5
*Cenostigma pyramidale*					–	–	–	–					–	12.5	–	12.5
*Centella* sp.	–	–	–	–									–	12.5	12.5	12.5
*Cissus* sp.													–	–	12.5	–
*Chorisia glaziovii*													–	12.5	12.5	12.5
*Cissampelos sympodialis*													–	–	–	25
*Cnidoscolus urens*	–	–	7.69	–	10	–	10	–					–	12.5	–	–
*Cnidoscolus quercifolius*					–	–	10	–	25	25	25	–	–	–	–	–
*Cocos nucifera*													–	12.5	–	–
*Combretum fruticosum*													–	–	12.5	–
*Copernicia prunifera*													–	12.5	–	–
*Croton* sp.					–	–	10	–					–	–	–	–
*Cymbopogon citratus*	–	–	–	–	–	10	–	–	25	–	–	–	–	–	–	–
*Cynophalla flexuosa*													–	12.5	12.5	–
*Erythrina velutina*	15.38	–	–	–	–	–	–	–					–	–	12.5	12.5
*Eucalyptus globulus*	–	–	–	–	–	–	–	–	–	–	–	–	–	–	–	–
*Foeniculum vulgare*	38.46	7.69	–	–	–	–	–	–	25	50	–	–	12.5	–	–	–
*Glycyrrhiza glabra*	–	–	–	7.69												
*Helianthus annuus*	–	–	–	–	–	–	10	–	–	–	25	–	–	–	–	–
*Hibiscus* sp.	7.69	–	–	–	–	–	–	10	–	–	–	–	–	12.5	–	–
*Hymenaea courbaril*	23.07	–	7.69	7.69	–	–	–	–	–	–	25	–	–	–	–	–
*Illicium verum*	15.38	–	7.69	7.69	–	–	30	10	–	–	–	–	–	–	–	–
*Lavandula angustifolia*	7.69	–	7.69	–	–	–	–	10	–	–	25	–	12.5	–	–	–
*Libidibia ferrea*	15.38	7.69	–	–	20	10	10	–					–	–	–	–
*Licania rigida*	15.38	15.4	–	–					25	–	–	–	12.5	–	12.5	–
*Lippia alba*	7.69	–	–	7.69	–	–	–	–					–	12.5	–	–
*Luffa operculata*	7.69	–	–	–	10	–	10	–					12.5	–	–	–
*Maytenus rigida*	–	–	–	–	–	–	–	–					–	–	12.5	–
*Mentha* sp.	–	–	–	–					25	25	25	–	–	–	–	–
*Miconia albicans*	–	–	7.69	7.69	–	–	10	–	–	–	–	–	–	–	–	–
*Monteverdia ilicifolia*	–	–	–	7.69	–	10	–	–	–	–	–	–	–	–	–	12.5
*Morus* sp.	7.69	–	–	7.69					–	–	–	–	–	–	–	–
*Musa* x *paradisíaca*													–	12.5	–	12.5
*Myracrodruon urundeuva*	–	–	–	–	30	10	20	–					–	–	–	–
*Persea americana*					–	–	–	–	–	–	–	–	–	–	–	12.5
*Phyllanthus niruri*	23.07	15.4	–	30.77	10	–	–	10	–	–	25	–	–	–	–	–
*Pimpinella anisum*	–	–	–	7.69	–	–	–	–	–	–	–	–	–	–	–	–
*Pombalia lanata*	–	–	23.08	–					–	–	–	–	–	12.5	25	12.5
*Pterodon emarginatus*	7.69	7.69	7.69	–	10	10	20	10	25	–	–	–	–	–	–	–
*Punica granatum*	7.69	–	–	–	10	–	–	–	–	–	–	–	–	–	–	25
*Rosmarinus officinalis*	–	–	7.69	–	10	–	–	–	–	–	–	–	12.5	12.5	12.5	–
*Salvia hispânica*	7.69	–	–	–	–	10	–	–	–	–	25	–	–	–	12.5	–
*Sambucus australis*	7.69	–	–	–	10	–	10	10	–	–	25	25	–	–	–	–
*Schnopsis brasiliensis*	–	–	–	–	–	10	10	10					–	–	12.5	12.5
*Senna* sp.	7.69	–	7.69	–	10	–	10	–	–	–	–	–	–	–	–	–
*Sesamum orientale*	–	–	–	–	10	–	–	–	25	25	25	–	–	–	12.5	25
*Sideroxylon obtusifolium*	–	–	–	–	–	10	10	10	–	–	25	–	–	–	–	–
*Solanum americanum*													–	12.5	–	–
*Symphytum officinale*	–	–	–	–									–	–	12.5	12.5
*Syzygium cumini*	–	–	–	–	–	–	–	–	–	–	–	–	–	12.5	–	–
*Tamarindus indica*	7.69	7.69	–	–									–	12.5	12.5	12.5
*Taraxacum officinale*	–	–	–	–					–	–	25	–	25	25	12.5	12.5
*Valeriana* sp.	–	7.69	–	–												
*Wilbrandia* sp.	–	–	–	7.69	–	–	–	–					–	–	–	–
*Ximenia americana*	–	–	–	–	–	–	–	–	–	–	25	–	–	–	–	–
*Zingiber officinale*	–	–	7.69	–	–	–	–	–	–	–	–	–	–	–	12.5	–
*Ziziphus joazeiro*	–	–	–	7.69									–	12.5	–	–

Two species deserve special attention in this scenario: (1) *Miconia albicans,* which in the beginning of the study was registered in few trade points, however, increased the frequency throughout the year, and was registered in many trade points at the end of the study, and (2) *F. vulgare*, which was cited by the traders as a common local cultivated species, but it was not observed in some moments of the year and, in this period, it had higher prices.

## Discussion

The medicinal plant species traded in the public markets of Paraíba were shared among the mesoregions, except for the Sertão, which had a more specific group of plants for sale. Climatic and seasonal variations did not seem to greatly influence plant availability throughout the year, considering that traders in general keep a stock of dry plants to ensure the supply of most species.

### Local knowledge and the medicinal plant trade

The highest relative importance values were recorded for *Punica granatum*, *Zingiber officinale*, and *Myracrodruon urundeuva*. Although these species were recorded in all studied mesoregions and kept a relatively frequent availability in the trading locations during the research, previous studies involving some of the studied municipalities did not record these species in the market. In Guarabira, *P. granatum* was not previously recorded among the main species, and *Z. officinale* and *M. urundeuva* were also not recorded in a previous study conducted in the market of Patos [24], even though *M. urundeuva* occurs naturally in the region [42, 43]. These data may indicate that over the past decade changes have occurred in plant availability or in the local importance of medicinal plants traded in these markets. It is worth mentioning that these species have been commonly documented in markets of nearby regions, such as in Pernambuco, although *P. granatum* and *Z. officinale* usually presented relatively low RI values compared to what was observed in our study [10, 18, 23].

The greater use of medicinal plants to treat diseases of the digestive and respiratory systems has often been reported in ethnobotanical studies [3, 14, 15, 21] and explained by the fact that these diseases are most commonly affecting the population [3, 15]. The emphasis on endocrine, nutritional, and metabolic diseases might partly be explained due to plant use for weight loss (Table 1), which, according to the traders, is also a consequence of the growing interest of customers in using plants that aid in losing weight and keeping a good shape. This has been related to the current habits of society, which tends to be sedentary and ingest highly caloric foods, becoming obese, and social media and television promoting the sale of medicinal plants for losing weight [44–46].

In most cases, the medicinal use of leaves, bark, and seeds (Fig. 3) was recorded, similar to several other studies [7, 30, 47]. Previously, a greater use of leaves and herbaceous plants in wetter regions, such as the Atlantic Forest, has been commonly registered [4, 48, 49], while in drier regions, such as the Caatinga, a predominance of the use of barks and woody plants has been shown [3, 50], highlighting a relationship with the loss of foliage in the vegetation during the drier periods [16]. This apparent correspondence between the most used plant parts and the environment can also be seen in other studies conducted in dry [6, 8, 51] and wetter environments [20, 33]. However, in the most cases of this study, it was not possible to establish a similar relationship between the environment and the most traded plant parts, and the Agreste was the only region where the bark was the main plant structure traded. The leaves were the main plant part in the remaining mesoregions, even in drier areas, where a more significant bark trade would have been expected. Agreste is an ecotone between dry and humid regions, despite the predominance of the semiarid area in this mesoregion. The bark commercialization in this mesoregion suggests a trade preference of the native species, and the bark can be obtained from tree species of the Caatinga (usually without leaves in the dry season). Cultural practices and the local knowledge heritance can support this use and the consumption of these species. A possible explanation for that is the dynamism of medicinal plant trade, which involves not only the local plant species in the studied markets but also species from other regions and even imported from other countries, since, as observed here, most species with high relative importance were exotic.

### Similarity between the mesoregions

Interestingly, the Agreste mesoregion, geographically located in a transition area between the Atlantic Forest and the Caatinga, fell in the center of the graph, sharing its limits with all remaining mesoregions. The intensity of trading of Atlantic and Caatinga species and the commercialized exotic species as well indicates that this region, as an ecotone, also favors the exchange of knowledge. This leads us to suggest that the Agreste mesoregion represents a not only as a transition area for vegetation, but also of knowledge and practice in the use of (medicinal) plants in comparison with the remaining mesoregions. In this context, besides being an important ecological area for conservation, the ecotone can also represent a region that demands attention for cultural proposes. This perspective could be an interesting guiding tool for future research involving the trade in medicinal plants.

Considering the relatively small territory of the state of Paraíba (56,585 km^2^) [35], it may be inferred that there is a permutation of knowledge and traded species between the different regions. Although a study with similarity analysis between different phytophysiognomies in the states of Paraíba and Pernambuco has not found similarity between the studied phytophysiognomies [52], it is worth noting that, in addition to the possibility of permutation of native species of different phytophysiognomies, the use of exotic species is a factor that collaborates for a greater sharing of species, even in so different areas as the Atlantic Forest and the Caatinga.

### Temporal variation in the medicinal plant trade

It was impossible to establish a relationship between the unavailability of any species during some period and the mesoregion, given that when one species was unavailable in one trade location (market stand), it could usually be found in other locations of the same market. While there might be a relationship between plant habit and availability, given most species that showed some period of unavailability are herbaceous, similar to other studies [10], most species are sold dried, giving the traders the possibility to simply acquire and stock the material to avoid a lack of the product [20], although at times traders simply might not have the financial resources to stock material.

In some cases, the informants stated that *Foeniculum vulgare,* according to them, cultivated in the Brejo and Curimataú areas (Agreste Mesoregion), was not available during some periods. A possible explanation for species unavailability in some cases could be traced to the recent sale of the whole stock by a trader, or the lack of interest by the trader in stocking a particular product given the low demand.

It was also possible to note the incorporation of a new species into the plant trade during our study: In the first stage of the interviews, *Miconia albicans* was only found in a few places of the Mata and Sertão mesoregions, and then, a fast spread of this species in the market was observed. Although being a native species and, according to some informants, common in woody areas of both the Atlantic Forest and the Caatinga, its medicinal use was not well known until recently. According to the traders, a recent increase in the trade of this species occurred due to their recent promotion to treat pain and muscular and rheumatic diseases on the internet and television, which led increasing to a consumer demand. Previous ethnobotanical studies did record this species, but made no reference to its medicinal use [53–55]. Similar cases have also been reported for *Hibiscus* sp., *Camellia sinensis*, and *Zingiber officinale*, species that, according to the informants, began to be traded less than a decade ago, also influenced by the media and the Internet. In the contemporary world, traditional medicinal practices have often incorporated other knowledge, e.g., biomedical knowledge, in the globalization process, in this way affecting selection and transmission mechanisms of knowledge [56].

## Conclusions

The inventory of medicinal plants available in the markets of Paraíba varies little throughout the year. In general, traders seem to keep permanent stocks of the main plants. Traders were also receptive to incorporating new plants into their stocks, which might be explained several factors, such as the influence of the media and the Internet, fostering the growing interest of customers in certain species.

It was impossible to establish a relationship between the periods of species absence in some trading locations and the mesoregion where this absence occurred. The absence during certain periods is probably more related to temporary unavailability or the impossibility for the trader to stock the product, or might even be related to environmental changes, which may influence species availability. It was not possible to establish a direct association between the climatic variations of each mesoregion and the plant part traded. The leaves were the more commercialized parts, even in the drier regions, except in the Agreste, an ecotone region, where the bark was the main part observed.

The Sertão mesoregion was the only one that showed a significant variation in the inventory of species sold by the traders. It is also interesting that the Agreste mesoregion, geographically located in an intermediate region between the Atlantic Forest and the Caatinga, showed an intermediate similarity pattern with the remaining mesoregions.

## Data Availability

The datasets used and/or analyzed during the current study are available from the corresponding author on reasonable request.
